# Impact of Visual Repetition Rate on Intrinsic Properties of Low Frequency Fluctuations in the Visual Network

**DOI:** 10.1371/journal.pone.0018954

**Published:** 2011-05-24

**Authors:** Yi-Chia Li, Chien-Chung Chen, Jyh-Horng Chen

**Affiliations:** 1 Graduate Institute of Biomedical Engineering and Bioinformatics, National Taiwan University, Taipei, Taiwan; 2 Interdisciplinary MRI/MRS Laboratory, Department of Electrical Engineering, National Taiwan University, Taipei, Taiwan; 3 Department of Psychology, National Taiwan University, Taipei, Taiwan; 4 Neurobiology and Cognitive Science Center, National Taiwan University, Taipei, Taiwan; Cuban Neuroscience Center, Cuba

## Abstract

**Background:**

Visual processing network is one of the functional networks which have been reliably identified to consistently exist in human resting brains. In our work, we focused on this network and investigated the intrinsic properties of low frequency (0.01–0.08 Hz) fluctuations (LFFs) during changes of visual stimuli. There were two main questions to be discussed in this study: intrinsic properties of LFFs regarding (1) interactions between visual stimuli and resting-state; (2) impact of repetition rate of visual stimuli.

**Methodology/Principal Findings:**

We analyzed scanning sessions that contained rest and visual stimuli in various repetition rates with a novel method. The method included three numerical approaches involving ICA (Independent Component Analyses), fALFF (fractional Amplitude of Low Frequency Fluctuation), and Coherence, to respectively investigate the modulations of visual network pattern, low frequency fluctuation power, and interregional functional connectivity during changes of visual stimuli. We discovered when resting-state was replaced by visual stimuli, more areas were involved in visual processing, and both stronger low frequency fluctuations and higher interregional functional connectivity occurred in visual network. With changes of visual repetition rate, the number of areas which were involved in visual processing, low frequency fluctuation power, and interregional functional connectivity in this network were also modulated.

**Conclusions/Significance:**

To combine the results of prior literatures and our discoveries, intrinsic properties of LFFs in visual network are altered not only by modulations of endogenous factors (eye-open or eye-closed condition; alcohol administration) and disordered behaviors (early blind), but also exogenous sensory stimuli (visual stimuli with various repetition rates). It demonstrates that the intrinsic properties of LFFs are valuable to represent physiological states of human brains.

## Introduction

Visual network is one of the functional networks which have been reliably identified to consistently exist in human resting brains [1], [2]. There have been many studies demonstrating the correlations between endogenous factor/behavior modulations and intrinsic properties of LFFs in this network. Yang et al. discovered significantly higher LFF power of visual cortices in EO (eye-open) than EC (eye-closed) condition during darkness [3]. Esposito et al. observed that alcohol induced resting-state functional connectivity of visual network, which may impair the normal activation response to visual stimuli and affect visual perception [4]. Yu et al. found decreased functional connectivity between primary visual area (PVA) and bilateral supplementary motor area (SMA) in early blind subjects [5]. Summarily, visual network is altered by modulations of both endogenous factors (EO, EC, and alcohol administration) and disordered behaviors (early blind).

In our study, we focused on the correlations between exogenous factor modulations and intrinsic properties of LFFs in visual network. We analyzed scanning sessions that contained rest and visual stimuli in various repetition rates with a novel method. The method included three numerical approaches involving Independent Component Analyses (ICA) [6], fractional Amplitude of Low Frequency Fluctuation (fALFF) [7], and coherence [8], to respectively investigate the modulations of visual network pattern, LFF power, and interregional functional connectivity across resting-state and visual stimuli at different repetition rates. There were two main questions to be discussed in this study: intrinsic properties of LFFs regarding (1) interactions between visual stimuli and resting-state; (2) impact of repetition rate of visual stimuli.

## Results

### Impact of Visual Repetition Rate on Visual Network Pattern

**Figure 1:**
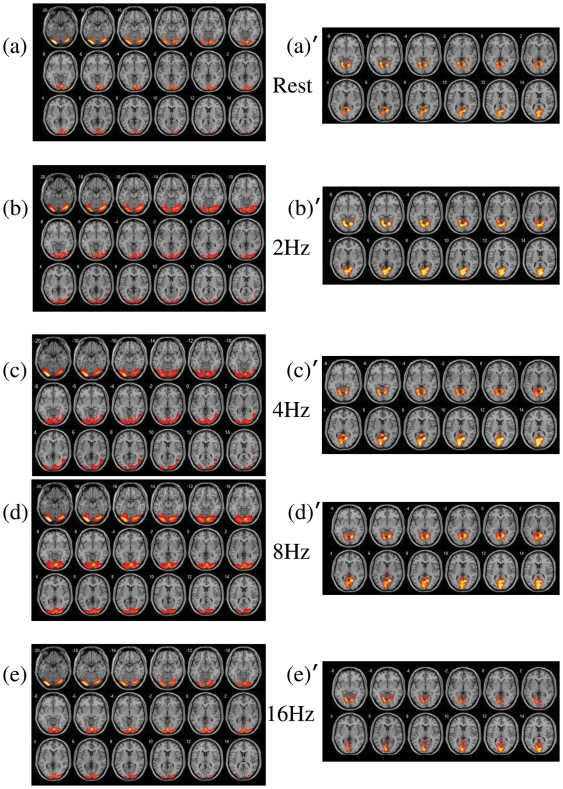
Impact of repetition rate on the patterns of two subsystems in visual network. (The number in left upper corner of each image represented the location of the image in z-direction of Talairach coordinates.)

We performed group ICA-based analyses to obtain visual networks during rest and visual stimulus rate = 2, 4, 8, 16 Hz. In Figure 1, (a)–(e) and (a)′–(e)′ respectively showed lateral and medial visual areas across various visual repetition rates. The lateral visual areas consisted of peristriate area and lateral and superior occipital gyrus (Brodmann area 19), whereas the medial visual areas predominantly consisted of striate and parastriate (Brodmann area 17/18). The two subsystems of visual network fitted in with prior literature [1] which illustrated that the visual network was divided into subsystems by ICA-based analyses.

By comparing Fig. 1(b)/(b)′ with Fig. 1(a)/(a)′, more areas of visual network were involved in visual processing when resting-state was replaced by visual stimuli. With increasing visual repetition rate, more areas of visual network were involved in visual processing, as Fig. 1(b)/(b)′–(d)/(d)′ shows. The maximum involved areas occurred in repetition rate = 8 Hz. When repetition rate was even higher, as Fig. 1(e)/(e)′ shows, less areas of visual network were involved in visual processing.

### Impact of Visual Repetition Rate on LFF power

**Figure 2:**
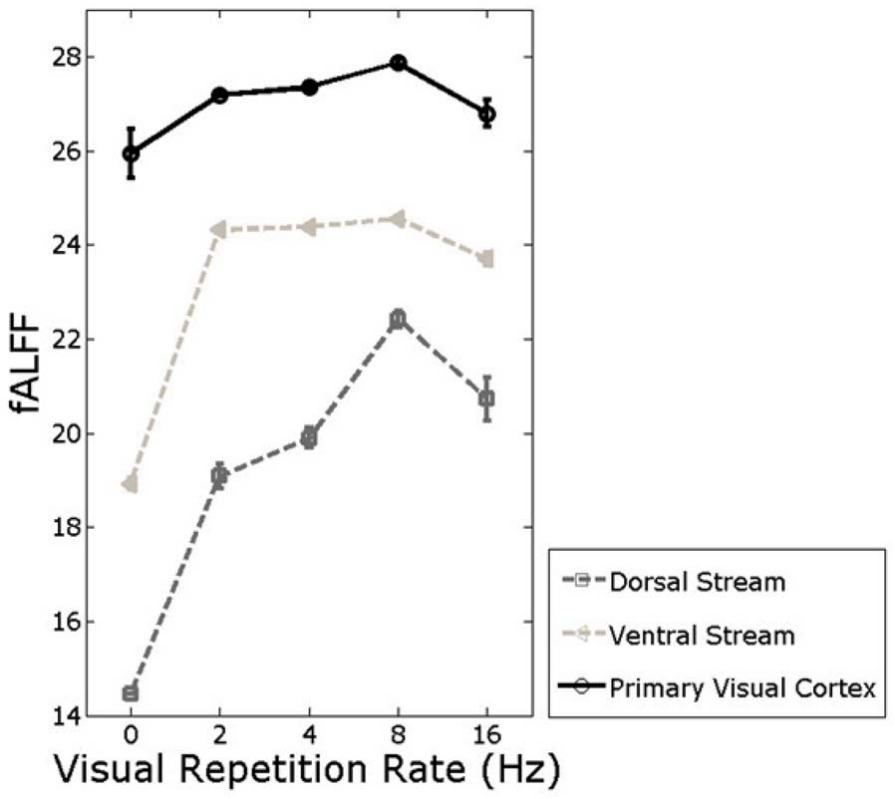
Impact of repetition rate on LFF power in three main areas of visual network.

Figure 2 showed the impact of visual repetition rate on LFF power in primary visual cortex, the dorsal stream, and the ventral stream. When visual repetition rate increased from 0 Hz to 2 Hz, larger fALFF occurred in all these three areas, which indicated that low frequency oscillations of visual network fluctuated more dramatically during visual stimuli rather than resting-state.

With higher repetition rate, fALFF in the three areas kept increasing. The largest fALFF occurred in repetition rate = 8 Hz. When repetition rate was even higher, fALFF decreased. This illustrated that low frequency oscillations of visual network fluctuated more dramatically with higher repetition rate. However, the low frequency fluctuations fluctuated the most dramatically in repetition rate = 8 Hz. When repetition rate was even higher, the low frequency oscillations fluctuated less dramatically.

### Impact of Visual Repetition Rate on Interregional Functional Connectivity

**Figure 3:**
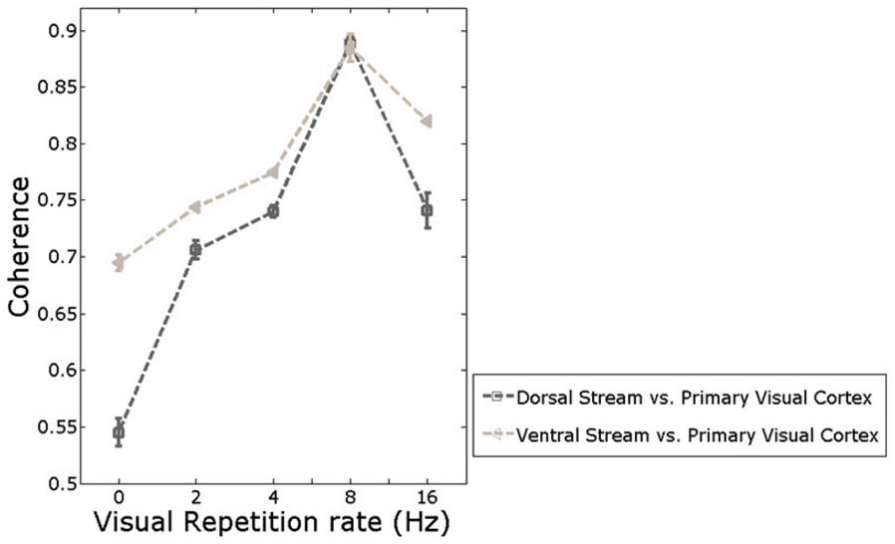
Impact of repetition rate on functional connectivity between two visual streams and primary visual cortex.

Figure 3 showed the impact of visual repetition rate on LFF coherence between two visual streams and primary visual cortex. When visual repetition rate increased from 0 Hz to 2 Hz, larger coherence occurred between both two visual streams and primary visual cortex, which represented higher consistency of LFFs between both two streams and primary visual cortex and indicated stronger interregional functional connectivity of visual network during visual stimuli rather than resting-state.

With higher visual repetition rate, coherence between both two visual streams and primary visual cortex kept increasing. The largest coherence occurred in repetition rate = 8 Hz. When the repetition rate was even larger, coherence decreased. This showed that higher repetition rate induced stronger interregional functional connectivity in visual network. However, the strongest interregional functional connectivity occurred in repetition rate = 8 Hz. When repetition rate was even higher, weaker interregional functional connectivity occurred.

## Discussion

### Why Did We Choose “Coherence” but not “Correlation” as the Index which Represented Interregional Functional Connectivity?

Compared with correlation analyses, coherence analyses are less sensitive to the shape of regional hemodynamic response function (HRF), which varies across regions due to interregional differences in vascular properties [8]. The insensitivity increases the accuracy of interregional functional connection measures. Therefore, coherence analyses were applied in our study as the index which represented interregional functional connectivity that was invariant to interregional differences in the HRF.

### Intrinsic Properties of LFFs Regarding Interactions between Visual Stimuli and Resting-state

Previous literatures demonstrated that inter-individual changes in resting-state, such as aging, gender, and even individual diversities, modified the response to a certain visual stimulus. Aging led to diminished top-down inhibitory control [9], decreased inter-hemispheric functional connectivity [10], and reduced functional activation [11] in response to visual stimuli, which may result in enhanced distractibility at the behavioral level for the elderly. Gender differences were found to highly influence focal activation in response to visual stimuli as well, which indicated stronger activation for women rather than men [12]. Furthermore, individual diversities affected their responses to the same visual stimulus. Baseline BOLD (Blood Oxygen Level Dependent) correlation was discovered to predict individuals' stimulus-evoked responses [13].

In our study, we used novel methods to focus on the investigation of the overall interactions between visual stimuli and resting-state without discussions on the specific related factors such as aging, gender, or individual diversities. According to the results of our work, when resting-state was replaced by visual stimuli, more areas of visual network participated in visual processing, the low frequency oscillations fluctuated more dramatically, and higher interregional functional connectivity occurred. Briefly, stronger interregional functional connections occurred in visual network during visual stimuli rather than resting-state.

### Intrinsic Properties of LFFs Regarding Visual Stimuli at Different Repetition Rates

In our study, we changed visual repetition rate to alter exogenous sensory stimulus conditions. With increasing visual repetition rate, more areas of visual network participated in visual processing, the low frequency oscillations fluctuated more dramatically, and higher interregional functional connectivity occurred. Briefly, stronger interregional functional connections occurred in visual network when repetition rate was higher. However, the strongest interregional functional connections occurred in repetition rate = 8 Hz. When the repetition rate was even higher, interregional functional connections became weaker. This corresponded to Briicke-Bartley Effects [14], [15], which described a periodic waxing and waning of the effectiveness to repeated stimuli, representing a period of 1/8 second. Since the repetition rate of the repeated stimuli was higher than 8 Hz, the channels of the human optic pathway would not recover rapidly enough to respond to the successive members of repeated stimuli.

In prior literatures, the relationship between temporal frequency of visual stimuli and biological signals measured by BOLD [16]–[18], rCBF (relative Cerebral Blood Flow) [19], EEG (Electroencephalography) [17], and MEG (Magnetoencephalography) [20], [21] was discussed. Corresponding to the results of our work that the strongest interregional functional connections occurred in repetition rate = 8 Hz, the maximum biological signals including BOLD responses, rCBF, EEG signals and MEG measurements also occurred at the temporal frequency of 8 Hz. It implied the high correlations between LFFs and these biological signals, which supported the hypothesized sources of LFFs including low frequency cardiovascular fluctuations [22], autoregulatory vasomotion [23], and changes of neuronal activity [24].

In our work, we investigated intrinsic properties of LFFs regarding impact of repetition rate using stimulation frequencies between 0–16 Hz. The interregional functional connections were found to peak at 8 Hz. In addition to around 8 Hz peak, previous literatures demonstrated that other peaks were observed for stimulation frequencies at around 20, 40, and 80 Hz [25]–[27]. These peaks might be due to neural oscillators, which preferably oscillate at certain frequencies, so called “resonance frequencies”. In another word, visual stimuli at those resonance frequencies are processed faster by human brains than the other frequencies. Furthermore, smaller BOLD responses to the other frequencies were indicated to be caused by a reduction of neuronal metabolic demand [28], which supported prior demonstrations.

### Factors Which Affect Intrinsic Properties of LFFs in Visual Network

Our study focused on the relationship between exogenous factor modulations (various visual repetition rates) and intrinsic properties of LFFs in visual network. According to prior discussions in our work, stronger interregional functional connections occurred in visual network when resting-state was replaced by visual stimuli. With changes of exogenous stimuli (visual repetition rates), interregional functional connections were also modulated. To combine the results of our work with prior literatures [3]–[5], intrinsic properties of LFFs in visual network are altered by the following factors: endogenous factors (EO and EC condition; alcohol administration), disordered behaviors (early blind), and exogenous sensory stimuli. It demonstrates that intrinsic properties of LFFs are valuable to represent physiological states of human brains.

## Materials and Methods

### Ethics Statements

1. The participant has no history of neurological disorder, medication use, substance abuse, or contraindications to MRI such as claustrophobia.
2. The participant has no metal implants such as a pacemaker or a stent graft.
3. The participant has no make-up or tattoo on the skin.
4. The procedures of this study have been approved by National Taiwan University Hospital, Taipei, Taiwan.
5. The participant understands exactly the procedures of the whole experiment and gives informed written consents to procedures before participation.

### Participants

Fourteen right-handed volunteers (7 M, 7 F), ranging from 20- to 32-years-old, were recruited, trained, and imaged. Handedness was determined by Edinburgh Handedness Inventory [29]. Score >+40 was used to ensure right handedness. The mean/std score of the fourteen volunteers was 90/15.19. None of the participants had the history of neurological disorder, medication use, substance abuse, or contraindications to MRI such as claustrophobia. All participants gave informed written consents to procedures approved by Medical Imaging Laboratory Research Ethics Board before participation.

### The Design of Experiment

**Figure 4:**
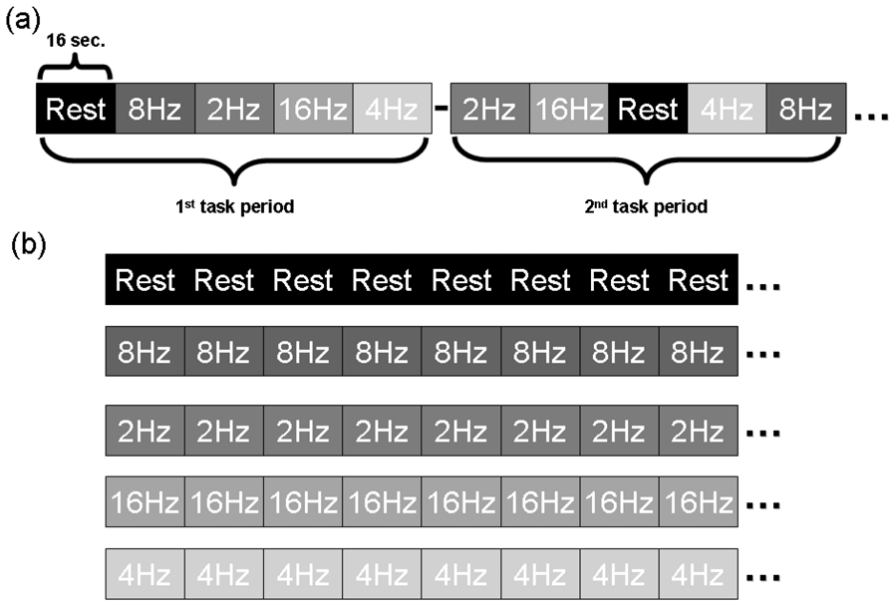
The design of experiment in our study.

Figure 4 (a) showed the design of experiment in our study. There were twelve task periods in the experiment. Each task period involved five stimulus conditions, which were rest and visual stimuli provided with checkerboard twinkling in the repetition rate = 2, 4, 8, and 16 Hz. In the rest condition, subjects were asked to face a screen without any visual stimuli and remain as still as possible, resting with their eyes open.

The five stimulus conditions in a task period were arranged in randomized order to prevent the results of our study from being affected by a regular pattern of stimuli. The duration of each stimulus condition within a task period was 16 s, which could avoid consistent and gradual reduction in activation caused by long duration of repeated visual stimuli (fMR adaptation) [30]. Hence, every task period which included five stimulus conditions lasted 80 s. The total time of the experiment which involved twelve task periods was 16 min. In addition, to avoid the situation that a participant did not concentrate on the visual stimuli during tasks, a tiny colorful cross randomly appeared in the center of the checkerboard. Participants in our study were requested to response by pressing the bell in their right hands to inform us they were still involved in the tasks.

After obtaining scanning sessions that contained rest and visual stimuli in various visual repetition rates, time series of the same stimulus condition in each task period were extracted and combined to become a new time series in a longer duration, as Figure 4 (b) showed. The five new time series of various stimulus conditions were processed in the subsequent analyses.

### The Location Determination of Primary Visual Cortex, Ventral Stream, and Dorsal Stream

In our study, we focused on three main regions of visual network including primary visual cortex, dorsal stream, and ventral stream and performed regional quantitative analyses to investigate intrinsic properties of LFFs in these regions during visual stimuli at different repetition rates. Particular fMRI experiments [31], [32] were applied to determine the locations of the three regions in the fourteen participants' brains.

**Figure 5:**
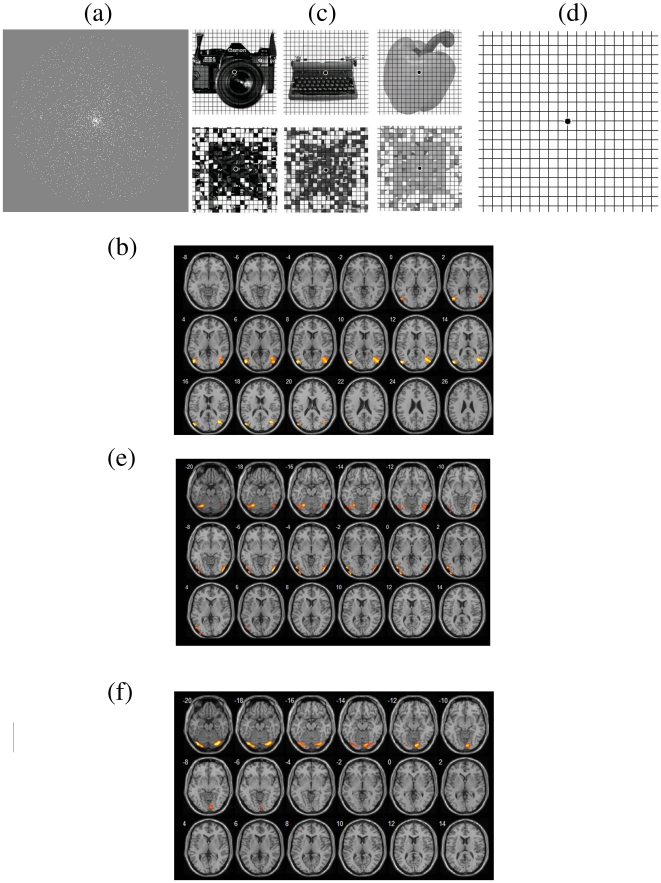
The location determination of primary visual cortex, ventral Stream, and dorsal Stream. (The number in left upper corner of each image represented the location of the image in z-direction of Talairach coordinates.)

Dorsal stream served spatial localization in visual system. Figure 5 (a) showed the picture which was shown to the participants in the task for the localization of dorsal stream. The picture consisted of many tiny white spots in the gray background. There were two stimulus conditions in the task, which were the spots freezing in the picture and the spots keeping moving radially back and forth in the picture. Each stimulus condition was 18 s in duration and appeared alternatively for six times. In the subsequent GLM (General Linear Model) activation group analysis, BOLD signals of the two stimulus conditions were compared to find the location of dorsal stream [33], [34], as Figure 5 (b) showed (p<0.001).

Figure 5 (c) and 5 (d) showed some examples of the pictures which were shown to the participants in the task for the localization of primary visual cortex and ventral stream. The upper row in Fig. 5 (c) displayed some examples of the object pictures whereas the bottom row exhibited some examples of the scrambled pictures which were formed by arranging the pixels of the object pictures in scrambled order. The contrast of the scramble pictures were the same as that of the object pictures. Fig. 5 (d) represented a black spot in the center of the background which was the same as the background of Fig. 5 (c).

There were three stimulus conditions in the task for the localization of primary visual cortex and ventral stream. In the first condition, the participants were asked to see the picture shown in Fig. 5 (d). In the second and third conditions, the object pictures and the scrambled pictures were respectively shown to the participants. Each object and scrambled picture appeared for 3 s in order to avoid visual fatigue to an individual picture. Each stimulus condition was 18 s in duration and appeared by turns for four times.

Ventral stream and primary visual cortex respectively served object recognition and receive and transmit visual information in visual system. In the subsequent GLM activation group analysis, BOLD signal time series of the scrambled pictures were compared with those of the object pictures to find the location of ventral stream [33], [34], as Figure 5 (e) showed (p<0.001). In addition, BOLD signal time series of a black spot in the center were compared with those of the scrambled pictures to find the location of primary visual cortex [33], [34], as Figure 5 (f) showed (p<0.001).

### Acquisition

Scanning was performed on a Bruker Medspec 3.0 Tesla whole body scanner. During the continuous scan, 480 24-slice whole-brain volumes were acquired using a T2*-weighted gradient-echo, echo-planar-imaging sequence (TR = 2,000 ms; TE = 30 ms; flip angle = 84°; slice thickness = 5 mm; slice gap = 0 mm; 4×4 mm^2^ in-plane voxel resolution; 64×64 image matrix, FOV 256×256 mm^2^). During the scans for the localization of primary visual cortex, dorsal stream, and ventral stream, the same imaging sequence and parameters were applied. However, 108 whole brain volumes were acquired in each scan for localizing an individual region.

At the end of fMRI data collection, fast spin-echo, 24–slice 2D T1- weighted anatomical images (TR = 2000 ms; TE = 17 ms; echo train length = 4; slice thickness = 5 mm; slice gap = 0 mm; 1×1 mm^2^ voxel resolution; 256×256 image matrix) were acquired in the same slice positions, in order to facilitate the precise determination of the structures corresponding to the functional activation foci.

### Preprocessing

**Figure 6:**
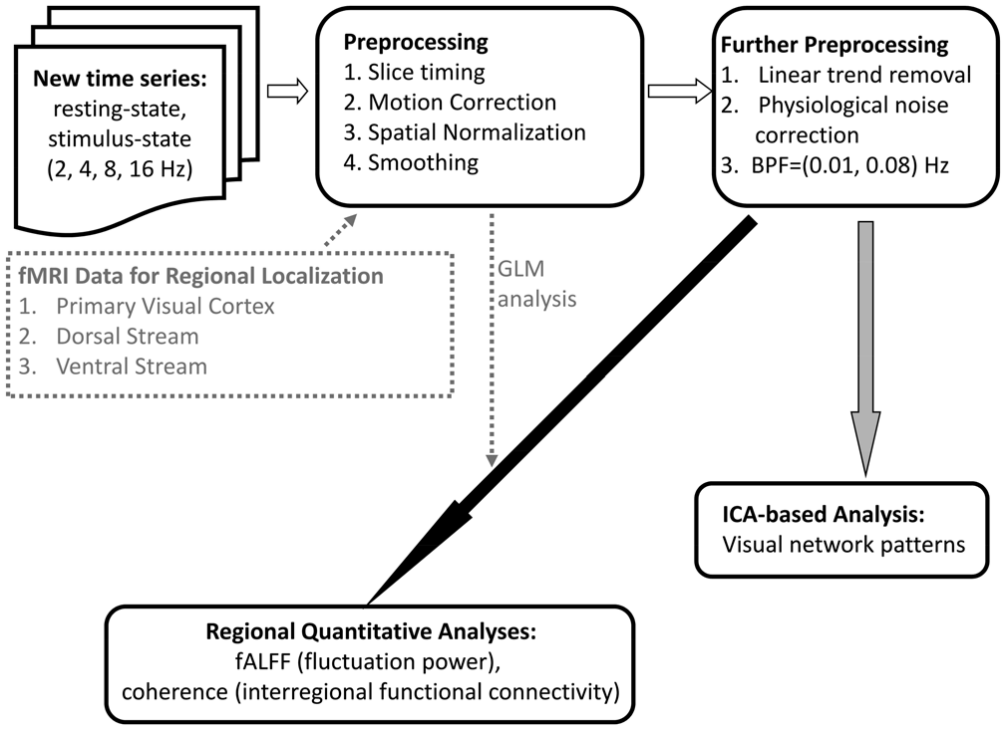
The flowchart of image processing in our study.

The five new time series of various stimulus conditions obtained in Fig. 4 (b) were processed based on the flowchart showed in Figure 6. GLM activation group analysis and preprocessing which contained slice timing, rigid body motion correction, spatial normalization, and Gaussian spatial smoothing (6 mm FWHM [35]) were performed using the SPM2 tool (http://www.fil.ion.ucl.ac.uk/spm).

Further preprocessing was performed using AFNI (analysis of functional neuroimaging, http://afni.nimh.nih.gov) software [36]. The further preprocessing included linear trend removal, correction of physiological noises, and temporally band-pass filtering (BPF; 0.01–0.08 Hz). Linear trend removal reduced linear drift of BOLD signals which were caused by imperfect shimming, nonlinearity and instability of gradient field, and loading effects of radiofrequency (RF) coils [37] during scan. AFNI-based version of PESTICA (http://www.nitrc.org/projects/pestica/) was applied to use temporal ICA-based analyses to determine cardiac and respiratory noises from fMRI data without parallel measurements of physiological signals [38]. PESTICA tool was also utilized for correcting the determined physiological noises from the data by RETROICOR (retrospective correction) method [39], in order to avoid respiratory and cardiac signals to be aliased into lower frequency band since the BOLD signal was sampled at a lower frequency (the Nyquist theorem). At the end of further preprocessing, temporally band-pass filtering (BPF; 0.01–0.08 Hz) was applied to obtain low frequency fluctuations only. The preprocessed new time series were applied in subsequent ICA-based analyses and regional quantitative analyses.

### ICA-based Analyses

ICA-based analyses implemented in the GIFT (Group ICA fMRI Toolbox; http://icatb.sourceforge.net/gift/gift_startup.php) were employed to perform a temporally concatenated group ICA on the preprocessed new time series to obtain visual networks during rest and visual stimuli in various repetition rates. We constrained the number of independent components to 20 [40]. After ICA decomposition we visually inspected all components and selected functional connectivity maps representing the visual network. Fig. 1 showed the visual networks during rest and various visual stimulus conditions.

### Regional Quantitative Analyses

In our work, we focused on the three main regions of visual network including primary visual cortex, dorsal stream and ventral stream and investigated intrinsic properties of LFFs in these three regions across different visual stimulus conditions. The numerical approaches involving fALFF [7] and regional coherence [8] were applied to help the investigation. Based on the locations of the three regions which were determined previously, the preprocessed new time series locating on these regions were selected for regional quantitative analyses.

In fALFF analyses, we calculated fALFF of the three main regions across different repetition rates, as Fig. 2 showed. The results represented the impact of visual repetition rate on low frequency fluctuation power in the three main regions of visual network. In coherence analyses, we calculated coherence of the time series between two visual streams and primary visual cortex across different repetition rates, as Fig. 3 showed. The results represented the impact of visual repetition rate on interregional functional connectivity between the two streams and primary visual cortex across different visual stimulus conditions.
